# Is it difficult to obtain inter-observer agreement in the measurement of the beta angle in ultrasound evaluation of the paediatric hip?

**DOI:** 10.1186/s13018-019-1263-1

**Published:** 2019-07-17

**Authors:** Ozgun Karakus, Ozgur Karaman, Ahmet Sinan Sari, Mehmet Mufit Orak, Hasan Hilmi Muratli

**Affiliations:** 1 Fatih Sultan Mehmet Training and Research Hospital, Omer Halisdemir University Hospital, Petrolıs st. Sümer bloc no: A-16 Kartal, İstanbul, Turkey; 2 Fatih Sultan Mehmet Training and Research Hospital, Istanbul, Turkey; 3 Omer Halisdemir University Hospital, Niğde, Turkey; 4 Fatih Sultan Mehmet Training and Research Hospital, Marmara University, Istanbul, Turkey

**Keywords:** Congenital hip dysplasia, Ultrasonography, Beta angle, Graf method

## Abstract

**Introduction:**

The aim of this study was to determine the differences and consistencies in the morphological and angular interpretations of standard USG images. Therefore, it was aimed to show the correlations of orthopaedic doctors with different periods of experience in hip ultrasound measurements taken with the Graf method.

**Materials and methods:**

The study included 210 infants randomly selected from those who presented at our hospital for DDH screening. A total of 6 ultrasound images were taken for each hip. These images were evaluated by  two paediatric orthopaedic professors, two orthopaedic specialists and two orthopaedic residents. The correlations of these measurements between all the doctors were evaluated statistically.

**Results:**

In beta angle evaluation, agreement between all the evaluators was at the level of 0.054. No agreement was seen between the two residents or between the two specialists (*p* = 0.003, *p* = 0.998, *p* = 0.998, respectively). Agreement between the two professors was determined at the level of 0.508 (*p* < 0.001). Agreement was determined at the level of 0.066 between the specialists and the residents. No agreement was observed between the specialists and the professors or between the professors and the residents (*p* = 0.014, *p* = 0.098, *p* = 0.737, respectively).

**Conclusions:**

It can be concluded that greater emphasis on the beta angle, the cartilage labrum, and more detailed explanations of this subject in the resident training program will achieve standardisation on this subject, and this is in direct proportion to clinical experience.

**Level of evidence:**

IV

## Introduction

Developmental dysplasia of the hip (DDH) is a clinical condition seen in a wide range varying from acetabular dysplasia which has disrupted the normal relationship between the femoral head and the acetabulum to a decentralised hip which has completely lost the joint relationship [1, 2]. DDH is the most commonly seen musculoskeletal system anomaly in childhood and, if not identified and treated, can lead to permanent deformities and arthrosis, also causing loss of workforce and reduced quality of life. Incidence has been reported as 0.7–20 per 1000 live births. The importance of early diagnosis in treatment has been proven, and in this respect, ultrasonography has become the currently recommended standard method [3, 4].

Hip ultrasonography is widely accepted as the primary method for screening, diagnosis and follow-up of treatment of DDH in newborns [5–9]. Various screening strategies have been recommended, one of which is neonatal ultrasound screening, as used in Germany, Switzerland and Australia [10, 11]. The most important factor determining the success of treatment is early diagnosis. Sequelae-free recovery is possible with treatment in the first months of life, while cases that are delayed can experience severe difficulties in treatment and permanent sequelae [12]. Ultrasonography (USG) is the most frequently used radiological method in the diagnosis and follow-up of DDH, and dysplasia which cannot be determined from physical examination and conventional radiography in the early period can be determined with USG [13].

The currently most widely used technique is the Graf static method. First, a coronal plane image is obtained and a qualitative evaluation is made of the hip bone and cartilage acetabular components and classification is made on the quantitative measurements between these components and the ilium. The alpha angle represents the bony roof of the acetabulum and the beta angle the cartilage roof. Ultrasonography is applied independently or as a part of a general clinical review of a date and physical examination [14]. An interesting finding reported by Bar-On et al. was that a hip evaluated as normal on ultrasound was only evaluated as normal on a repeated evaluation at the rate of 98%. This is because the ultrasound measurement is a screening test dependent on the person making the evaluation [15]. Differences in interpretation may be due to the specialist making the USG examination. In the Graf method, specific markers are used to reduce interpretation differences. These markers are the vertical iliac wing image, the deepest point of the acetabulum and the labrum. Nevertheless, however standardised the method, differences are still experienced in the evaluation of USG images.

The aim of this study was to determine the differences and consistencies in the morphological and angular interpretations of standard USG images. Therefore, it was aimed to show the correlations of orthopaedic doctors with different periods of experience in hip ultrasound measurements taken with the Graf method.

## Material and methods

The study included 210 infants randomly selected from those who presented at our hospital for DDH screening. The infants comprised 114 males and 96 females with a mean age of 11 weeks (range, 3–21 weeks). All the measurements were taken on a Sonoline G60S® ultrasound system (SIEMENS, Erlangen, Germany), using a 7.5-MHz linear probe [16]. All the infants were examined by the same doctor then laid in a lateral position on the USG table with the hip to be evaluated uppermost. Each hip was evaluated separately with only the doctor, the patient and the patient’s mother in the examination room. A total of six ultrasound images were taken for each hip.

These images were evaluated by two paediatric orthopaedic professors, two orthopaedic specialists and two orthopaedic residents. All the raters were blinded to the evaluation results of the others. All had completed a training program on the use of hip ultrasound with the Graf method. The measurements were taken with a goniometer according to the Graf method [17]. Each doctor evaluated 210 ultrasound images in respect of whether on each hip ultrasound image, there was a measurement that could be evaluated and whether or not it was in the standard plane and the measurements of the alpha angle, beta angle, hip type, anatomic verification, bone roof status (angled, curved or straight), cartilage roof status (present or absent) and morphological evaluation (mature or immature). The correlations of these measurements between all the doctors were evaluated statistically.

### Statistical analyses

Data obtained in the study were analysed statistically using NCSS 2007 software (Number Cruncher Statistical System, Kaysville, Utah, USA). When evaluating the study data, descriptive statistical methods were used (mean, standard deviation, frequency, percentage). In the determination of the levels of compatibility related to anatomic verification; standard plane, which the image can be evaluated; bone roof; cartilage roof; morphological evaluation; and the type of hip, Gwet’s AC1 was used. In the determination of the levels of compatibility related to alpha and beta angle variables, ICC was used. A value of *p* < 0.05 was accepted as statistically significant (Table 1).

**Table 1:** Cicchetti DV. Guidelines, criteria and rules of thumb for evaluating normed and standardised assessment instruments in psychology. Psychological Assessment. 1994;6(4):284–290

ICC	Comment
< 0.40	Poor
0.40–0.59	Fair
0.60–0.74	Good
0.75–1.00	Excellent

## Results

Evaluation was made of the USG images of 210 randomly selected infants comprising 114 males and 96 females. Compatibility of the evaluations was examined in paired groups for the two residents, the two specialists and the two paediatric orthopaedic professors.

### Anatomic verification

Agreement between all the evaluators was at the level of 0.768 and was determined as 0.887 between the two residents, 0.462 between the two specialists and 0.898 between the two professors (*p* < 0.001, *p* < 0.001, *p* < 0.001, *p* < 0.001, respectively).

### Standard plane evaluation

Agreement between all the evaluators was at the level of 0.924 and was determined as 0.915 between the two residents, 0.887 between the two specialists and 0.965 between the two professors (*p* < 0.001, *p* < 0.001, *p* < 0.001, *p* < 0.001, respectively).

### The USG image can be evaluated

Agreement between all the evaluators was at the level of 0.890 and was determined as 0.883 between the two residents, 0.777 between the two specialists and 0.970 between the two professors (*p* < 0.001, *p* < 0.001, *p* < 0.001, *p* < 0.001, respectively).

### Bone roof evaluation

Agreement between all the evaluators was at the level of 0.388 and was determined as 0.219 between the two residents, 0.264 between the two specialists and 0.531 between the two professors (*p* < 0.001, *p* < 0.001, *p* < 0.001, *p* < 0.001, respectively).

### Cartilage roof evaluation

Agreement between all the evaluators was at the level of 0.905 and was determined as 0.796 between the two residents, 0.955 between the two specialists and 0.964 between the two professors (*p* < 0.001, *p* < 0.001, *p* < 0.001, *p* < 0.001, respectively).

### Morphological evaluation

Agreement between all the evaluators was at the level of 0.813 and was determined as 0.795 between the two residents, 0.712 between the two specialists and 0.818 between the two professors (*p* < 0.001, *p* < 0.001, *p* < 0.001, *p* < 0.001, respectively).

### Alpha angle evaluation

**Fig. 1:**
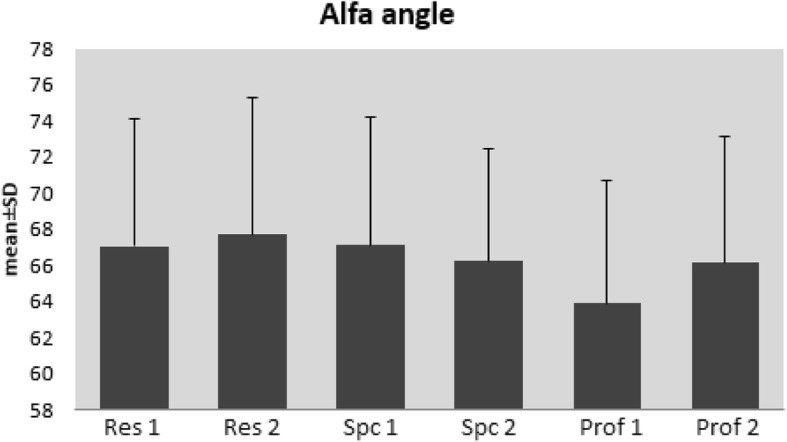
Distributions according to the alpha angle

Agreement between all the evaluators was at the level of 0.622 and was determined as 0.470 between the two residents, 0.651 between the two specialists and 0.675 between the two professors (*p* < 0.001, *p* < 0.001, *p* < 0.001, *p* < 0.001, respectively) (Fig. 1).

### Beta angle evaluation

**Fig. 2:**
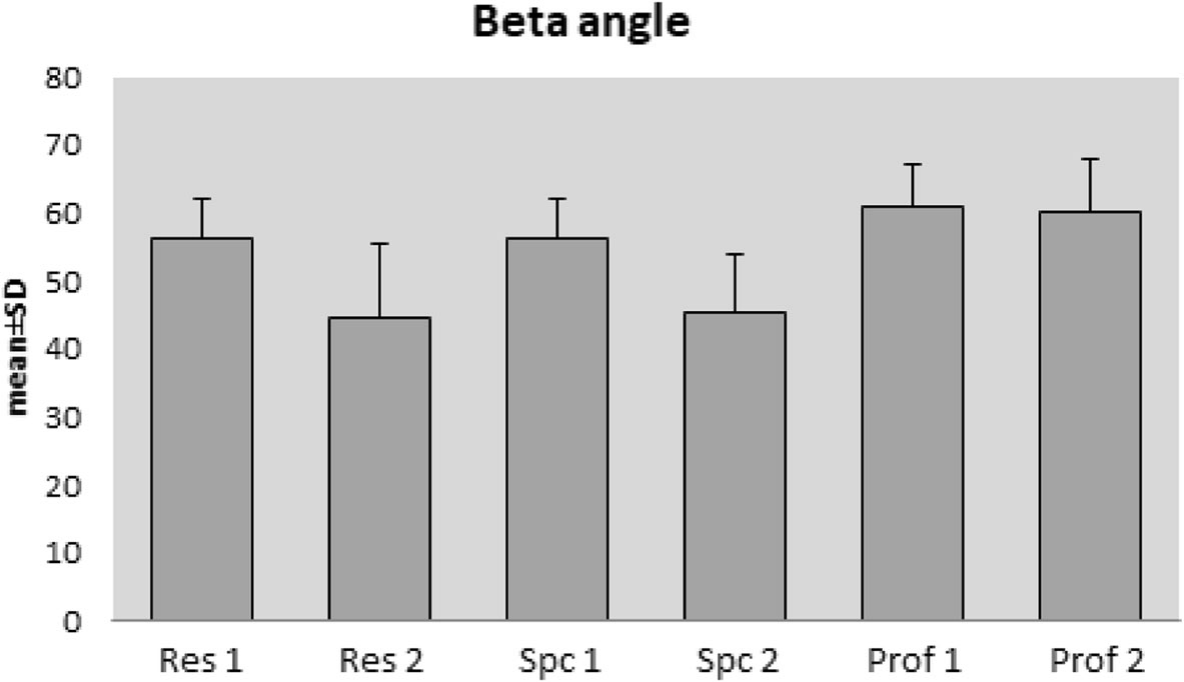
Distributions according to the beta angle

Agreement between all the evaluators was at the level of 0.054. No agreement was seen between the two residents or between the two specialists (*p* = 0.003, *p* = 0.998, *p* = 0.998, respectively). Agreement between the two professors was determined at the level of 0.508 (*p* < 0.001) (Fig. 2).

### Type evaluation

**Fig. 3:**
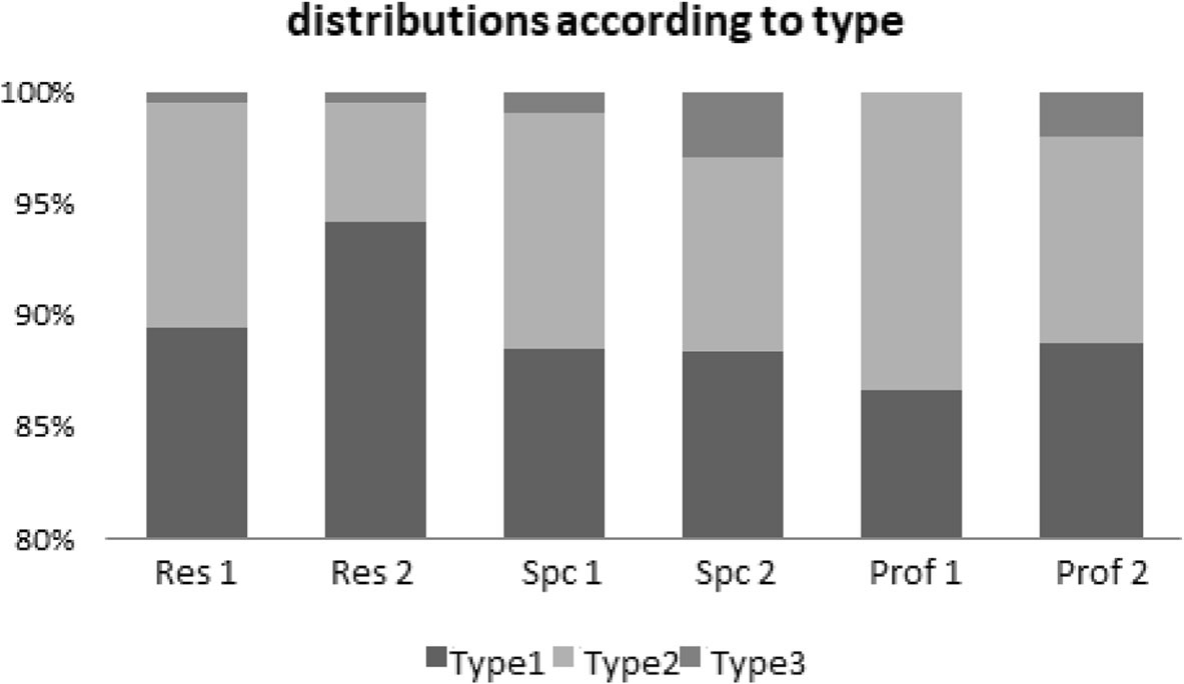
Distributions according to the types

Agreement between all the evaluators was at the level of 0.893 and was determined as 0.910 between the two residents, 0.892 between the two specialists and 0.866 between the two professors (*p* < 0.001, *p* < 0.001, *p* < 0.001, *p* < 0.001, respectively) (Fig. 3). All the values are presented in Table 2. Agreement levels were then examined between the residents, specialists and paediatric orthopaedic professors.

**Table 2:** Levels of agreement between the specialists, residents and professors and in the paired groups, related to the variables

	All (Prof-specialist-resident)	Resident 1 vs 2	Specialist 1 vs 2	Prof 1 vs 2
Anatomic verification	0.768 (0.726, 0.810), *p* < 0.001**	0.887 (0.837, 0.938), *p* < 0.001**	0.462 (0.361, 0.563), *p* < 0.001**	0.898 (0.853, 0.944), *p* < 0.001**
Standard plane	0.924 (0.899, 0.949), *p* < 0.001**	0.915 (0.874, 0.955), *p* < 0.001**	0.887 (0.840, 0.934), *p* < 0.001**	0.965 (0.938, 0.993), *p* < 0.001**
The USG can be evaluated	0.890 (0.861, 0.919), *p* < 0.001**	0.883 (0.835, 0.931), *p* < 0.001**	0.777 (0.701, 0.853), *p* < 0.001**	0.970 (0.945, 0.996), *p* < 0.001**
Bone roof	0.388 (0.342, 0.434), *p* < 0.001**	0.219 (0.134, 0.305), *p* < 0.001**	0.264 (0.174, 0.355), *p* < 0.001**	0.531 (0.437, 0.625), *p* < 0.001**
Cartilage roof	0.905 (0.879, 0.931), *p* < 0.001**	0.796 (0.733, 0.859), *p* < 0.001**	0.955 (0.922, 0.988), *p* < 0.001**	0.964 (0.928, 0.999), *p* < 0.001**
Morphological evaluation	0.813 (0.773, 0.853), *p* < 0.001**	0.795 (0.734, 0.856), *p* < 0.001**	0.712 (0.640, 0.784), *p* < 0.001**	0.818 (0.752, 0.884), *p* < 0.001**
Type	0.893 (0.858, 0.928), *p* < 0.001**	0.910 (0.865, 0.955), *p* < 0.001**	0.892 (0.833, 0.950), *p* < 0.001**	0.866 (0.809, 0.923), *p* < 0.001**
Alpha angle	0.622 (0.566, 0.678), *p* < 0.001**	0.470 (0.356, 0.570), *p* < 0.001**	0.651 (0.564, 0.724), *p* < 0.001**	0.675 (0.592, 0.744), *p* < 0.001**
Beta angle	0.054 (0.015, 0.103), *p* = 0.003**	− 0.204 (− 0.332, − 0.070), *p* = 0.998	−0.203 (−0.331, −0.067), *p* = 0.998	0.508 (0.398, 0.604), *p* < 0.001**

For anatomic verification; standard plane, which the image can be evaluated; bone roof; cartilage roof; morphological evaluation; and type variables, Gwet’s AC1 was calculated. For alpha and beta angle variables, ICC was calculated

**p < 0.01

### Anatomic verification

Agreement was determined at the level of 0.688 between the specialists and professors, 0.705 between the specialists and the residents and 0.898 between the professors and the residents (*p* < 0.001, *p* < 0.001, *p* < 0.001, respectively).

### Standard plane evaluation

Agreement was determined at the level of 0.922 between the specialists and professors, 0.911 between the specialists and the residents and 0.939 between the professors and the residents (*p* < 0.001, *p* < 0.001, *p* < 0.001, respectively).

### The USG image can be evaluated

Agreement was determined at the level of 0.883 between the specialists and professors, 0.860 between the specialists and the residents and 0.924 between the professors and the residents (*p* < 0.001, *p* < 0.001, *p* < 0.001, respectively).

### Bone roof evaluation

Agreement was determined at the level of 0.470 between the specialists and professors, 0.335 between the specialists and the residents and 0.335 between the professors and the residents (*p* < 0.001, *p* < 0.001, *p* < 0.001, respectively).

### Cartilage roof evaluation

Agreement was determined at the level of 0.950 between the specialists and professors, 0.880 between the specialists and the residents and 0.883 between the professors and the residents (*p* < 0.001, *p* < 0.001, *p* < 0.001, respectively).

### Morphological evaluation

Agreement was determined at the level of 0.789 between the specialists and professors, 0.807 between the specialists and the residents and 0.823 between the professors and the residents (*p* < 0.001, *p* < 0.001, *p* < 0.001, respectively).

### Alpha angle evaluation

Agreement was determined at the level of 0.660 between the specialists and professors, 0.641 between the specialists and the residents and 0.573 between the professors and the residents (p < 0.001, *p* < 0.001, *p* < 0.001, respectively).

### Beta angle evaluation

Agreement was determined at the level of 0.066 between the specialists and the residents. No agreement was observed between the specialists and the professors or between the professors and the residents (*p* = 0.014, *p* = 0.098, *p* = 0.737, respectively).

### Evaluation of hip type

Agreement was determined at the level of 0.877 between the specialists and professors, 0.917 between the specialists and the residents and 0.886 between the professors and the residents (*p* < 0.001, *p* < 0.001, *p* < 0.001, respectively). All the results are shown in Table 3.

**Table 3:** Levels of agreement between the specialists, residents and professors related to the variables

	Specialist vs Prof	Specialist vs resident	Prof vs resident
Anatomic verification	0.688 (0.630, 0.745), *p* < 0.001**	0.705 (0.651, 0.756), *p* < 0.001**	0.898 (0.868, 0.928), *p* < 0.001**
Standard plane	0.922 (0.894, 0.950), *p* < 0.001**	0.911 (0.881, 0.941), *p* < 0.001**	0.939 (0.915, 0.963), *p* < 0.001**
The USG can be evaluated	0.883 (0.850, 0.916), *p* < 0.001**	0.860 (0.822, 0.898), *p* < 0.001**	0.924 (0.898, 0.950), *p* < 0.001**
Bone roof	0.470 (0.416, 0.524), *p* < 0.001**	0.335 (0.285, 0.386), *p* < 0.001**	0.335 (0.285, 0.386), *p* < 0.001**
Cartilage roof	0.950 (0.925, 0.975), *p* < 0.001**	0.880 (0.847, 0.913), *p* < 0.001**	0.883 (0.848, 0.917), *p* < 0.001**
Morphological evaluation	0.789 (0.742, 0.836), *p* < 0.001**	0.807 (0.763, 0.851), *p* < 0.001**	0.823 (0.780, 0.866), *p* < 0.001**
Type	0.877 (0.837, 0.917), *p* < 0.001**	0.917 (0.882, 0.951), *p* < 0.001**	0.886 (0.850, 0.922), *p* < 0.001**
Alpha angle	0.660 (0.601, 0.717), *p* < 0.001**	0.641 (0.580, 0.699), *p* < 0.001**	0.573 (0.506, 0.638), *p* < 0.001**
Beta angle	0.038 (− 0.019, 0.105), *p* = 0.098	0.066 (0.007, 0.134), *p* = 0.014*	− 0.019 (− 0.069, 0.041), *p* = 0.737

For anatomic verification; standard plane, which the image can be evaluated; bone roof; cartilage roof; morphological evaluation; and type variables, Gwet’s AC1 was calculated. For alpha and beta angle variables, ICC was calculated

*p < 0.01. **p < 0.01

## Discussion

Developmental dysplasia of the hip (DDH) is the general term for a wide range of anatomic disorders of different degrees such as teratologic, unstable, subluxated, dislocated hip and acetabular dysplasia which is congenital or can develop in the postnatal period [18]. The incidence of DDH shows great differences according to ethnicity and geographic regions. In Europe, the incidence has been reported as 1–5.2%, and in Turkey, the rate has been determined as 1–1.5%. The most important factor determining treatment success is early diagnosis [19].

Hip USG was developed by Graf for the early diagnosis of DDH, became widely accepted in subsequent years, and started to be used in many countries in DDH screening [20, 21]. Several studies have demonstrated the success of USG screening in the early diagnosis of DDH. Especially in silent dysplasia, sonographic screening is the most important tool. Dysplastic hips that cannot be determined with physical examination can be revealed ultrasonographically [22]. This shows the importance of standardising USG screening. USG measurements are dependent on the evaluator, and discrepancies in measurements may be due to variability in the USG examination itself or in its interpretation. Studies have demonstrated that both the performance of USG and its interpretation influence the results and potential treatment [23].

After completion of all the measurements in the current study, each group was compared in pairs.

In respect of anatomic verification, the highest level of agreement was between the two paediatric orthopaedic professors (0.898) and the lowest level of agreement was between the two specialists (0.462).

In respect of the standard plane evaluation, the highest level of agreement was between the two paediatric orthopaedic professors (0.965) and the lowest level of agreement was between the two specialists (0.887).

When the USG image was examined in respect of whether it could be evaluated, the highest level of agreement was between the two paediatric orthopaedic professors (0.970) and the lowest level of agreement was between the two specialists (0.777).

In respect of the bone roof status, the highest level of agreement was between the two paediatric orthopaedic professors (0.531) and the lowest level of agreement was between the two residents (0.219).

In respect of the cartilage roof status, the highest level of agreement was between the two paediatric orthopaedic professors (0.964) and the lowest level of agreement was between the two residents (0.796).

When the images were examined in respect of morphology, the highest level of agreement was between the two paediatric orthopaedic professors (0.818) and the lowest level of agreement was between the two specialists (0.712).

In respect of the alpha angle measurement, the highest level of agreement was between the two paediatric orthopaedic professors (0.675) and the lowest level of agreement was between the two residents (0.470).

In respect of the beta angle measurement, the highest level of agreement was between the two paediatric orthopaedic professors (0.508) but no agreement could be determined between the two specialists or between the two residents (*p* = 0.998, *p* = 0.003, respectively).

When the USG images were examined in respect of hip type, the highest level of agreement was between the two residents (0.910) and the lowest level of agreement was between the paediatric orthopaedic professors (0.866).

With the exception of hip-type evaluation, it was clear that the greatest levels of agreement in a general sense were between the two paediatric orthopaedic professors. Each was blinded to the values of the other, but on 210 hip USG images, the evaluations were almost completely the same. Thus, it was clearly seen that standardisation was achieved when evaluating the USG images in direct proportion to the years of experience. However, in the evaluation of the hip type, the agreement between the two paediatric orthopaedic professors was low. This was thought to be related to the detailed hip examination made by the professors and the years of experience. Borderline angle values could have affected this result.

In the evaluation of the beta angle, no statistical agreement was determined between the residents or between the specialists. This demonstrated the importance of correct evaluation of the beta angle, in other words, the cartilage labrum. While no agreement was found for the beta angles, the greatest level of agreement in the hip-type evaluation was between the residents. This was a surprising result and could be explained by the fact that in evaluating the hip type, the beta angle is important especially in the differentiation of type 2C and type D hips. As there were relatively few type 2C and type D hips, this could explain the highest level of agreement seen in hip-type evaluation, while there was no agreement in the evaluation of the beta angles.

When the levels of agreement are examined in general between the paediatric orthopaedic professors, specialist doctors and resident doctors, in respect of anatomic verification, the highest level of agreement was seen between the professors and the residents (0.898) and the lowest level of agreement between the professors and the specialists (0.688).

In respect of the standard plane, the highest level of agreement was seen between the professors and the residents (0.939) and the lowest level of agreement between the specialists and the residents (0.911).

When the USG images were examined in respect of whether they could be evaluated, the highest level of agreement was seen between the professors and the residents (0.924) and the lowest level of agreement between the specialists and residents (0.860).

In respect of the bone roof, the highest level of agreement was seen between the professors and the specialists (0.470). The agreement levels between the professors and the residents and between the specialists and the residents were determined to be equal (0.335).

In respect of the cartilage roof, the highest level of agreement was seen between the professors and the specialists (0.950) and the lowest level of agreement between the specialists and the residents (0.880).

In respect of morphological evaluation, the highest level of agreement was seen between the professors and the residents (0.823) and the lowest level of agreement between the professors and the specialists (0.789).

In respect of the alpha angle, the highest level of agreement was seen between the professors and the specialists (0.660) and the lowest level of agreement between the professors and the residents (0.573).

In respect of the beta angle, while there was an agreement between the specialists and the residents (0.880), no agreement was observed between the professors and the specialists or between the professors and the residents (*p* = 0.098, *p* = 0.737, respectively).

When the USG images were examined in respect of hip type, the highest level of agreement was seen between the specialists and the residents (0.917) and the lowest level of agreement between the professors and the specialists (0.877).

When these results were evaluated taking as reference the hip USG evaluations of the two paediatric orthopaedic professors, who were both executive board members of the Paediatric Orthopaedic Association, it was aimed to see how close to these results the measurements were of the specialist and resident doctors. While there was greater agreement between the residents and the professors in respect of anatomic verification, which the USG image could be evaluated and the morphological evaluation, the agreement between the specialists and the professors was greater in respect of the bone roof, cartilage roof and the alpha angle evaluations.

In the evaluation of the beta angle, there was no agreement of the measurements between the paediatric othopaedic professors and the specialists or the residents. This demonstrated that standardisation was not achieved in the evaluation of the beta angle and the cartilage labrum was not sufficiently understood. The data clearly showed the necessity for the focus to be on this subject in training for paediatric orthopaedic hip USG evaluation.

Previous studies in literature have shown a correlation of USG evaluation results. In a study by Simon et al., USG agreement according to Graf classifications was evaluated between the radiology team, orthopaedic specialists, residents and paediatricians [24]. The highest levels of agreement were obtained between the paediatricians and the orthopaedic specialists. The authors attributed this to the experience of the doctors based on many years of ultrasound. In contrast to previous results, in the current study, the three researchers analysed both USG performed on newborns and their own results. No statistically significant difference could be found between the measurements of the researchers. This result was not anticipated as the paediatric orthopaedic surgeons perform almost a thousand hip USG examinations a year, but this number of hip USG measurements is not possible for the residents.

In the current study, the resident doctors who evaluated the USG images were selected from senior residents who had nearly finished their specialist training. These resident doctors, who had successfully passed the paediatric hip USG training, had taken the course and received certification in all paediatric hip USG evaluation. Therefore, with the exception of the beta angle, there was an agreement with each other in almost all the parameters. However, evaluation of the beta angle was seen to be directly related to years of experience.

The reason for the general disagreement and paired disagreement between the specialist orthopaedic doctors was considered to be that a long period of time had passed since they had taken the Graf hip USG training programme and it had not been repeated during the specialisation period. Moreover, the resident doctors applied what had been directly learned without interpreting the information given, whereas the specialist doctors did not apply the information given without interpreting it according to their clinical experience, and this affected the results.

In another study by Roovers et al. [25], the examinations were performed by diagnostic radiographers under the supervision of the project team (radiologist, orthopaedic resident, orthopaedic surgeon and child health-care physician) and the inter- and intra-observer agreements in the hip ultrasound evaluations were satisfactory for screening purposes. Although the inter-observer agreement was slightly low, it was concluded that if the researchers were well-trained, hip ultrasound evaluation had good potential as a screening tool for DDH. As shown in the current study, standardisation is very important in hip evaluation with the Graf method, and this can be achieved with regular hip USG training programmes and experience.

## Conclusion

The results of this study revealed the importance of the paediatric hip USG application and evaluation courses, which are organised by the Paediatric Orthopaedic Association and are compulsory for all orthopaedic resident doctors. That it is important to obtain standardisation and that this will increase with experience is shown in the evaluation of paediatric hip USG images. It is clear that a specialist or resident evaluating 200 paediatric hip USG images per year does not have the potential for the same evaluation as a paediatric orthopaedic professor evaluating more than a thousand USG images per year.

In a general sense, the measurements of all the evaluators were seen to be correlated with each other. With the exception of the beta angle, most of the parameters were in agreement and the values were significant. This is an indicator of sufficient experience and the success obtained from the hip ultrasound course programmes. However, the beta angle was revealed as the parameter on which there should be the main focus. It can be concluded that greater emphasis on the beta angle, the cartilage labrum, and more detailed explanations of this subject in the resident training programme will achieve standardisation on this subject, and this is in direct proportion to clinical experience.

## Data Availability

Applicable.

## Abbreviations

DDH: Developmental dysplasia of the hip
Prof: Professor
USG: Ultrasound
